# Validation of MRI for Volumetric Quantification of Atelectasis in the Perioperative Period: An Experimental Study in Swine

**DOI:** 10.3389/fphys.2019.00695

**Published:** 2019-06-04

**Authors:** Eric Noll, Mickael Ohana, Maryse Hengen, Elliott Bennett-Guerrero, Michele Diana, Celine Giraudeau, Julien Pottecher, Nicolas Meyer, Pierre Diemunsch

**Affiliations:** ^1^Institut Hospitalo-Universitaire “Image-Guided Surgery”, Université de Strasbourg, Strasbourg, France; ^2^Department of Anesthesia and Intensive Care, Strasbourg University Hospital, Strasbourg, France; ^3^Fédération de Médecine Translationnelle de Strasbourg (FMTS), Faculté de Médecine, Institut de Physiologie, Université de Strasbourg, Strasbourg, France; ^4^Department of Radiology, Strasbourg University Hospital, Strasbourg, France; ^5^Department of Anesthesiology, Stony Brook Medicine, Stony Brook, NY, United States; ^6^Department of Public Health, Groupe Methodes en Recherche Clinique (GMRC), Strasbourg University Hospital, Strasbourg, France; ^7^iCube, UMR7357, University of Strasbourg, Strasbourg, France

**Keywords:** lung imaging, perioperative medicine, postoperative pulmonary complications, lung physiopathology, alveolar recruitment

## Abstract

**Background:** Impairment of pulmonary aeration is a frequent postoperative complication that is associated with adverse outcome. Diagnosis and quantification of impaired pulmonary aeration by CT scan is limited due to concern for exposure to ionizing radiation. Magnetic resonance imaging (MRI) represents a potential radiation-free alternative for this use. We undertook an experimental study to validate the use of MRI to quantify pulmonary aeration impairment.

**Methods:** Ten *large white* pigs were studied before intubation, after intubation, 2 h after non-protective mechanical ventilation and after intra-tracheal negative pressure suction to induce atelectasis. A lung CT scan immediately followed by a lung MRI were performed at all four time points. On the 40 CT images lung volumes corresponding to non-aerated, poorly aerated, normally aerated, and overinflated voxels were measured based on their radiodensity. Similarly, on the 40 MRI images lung volumes corresponding to non-aerated and aerated voxels were measured based on their signal intensity. The correlation between non-aerated lung by MRI vs., CT scans, and with PaO_2_/FiO_2_ measured at each of the four time points was assessed with the Pearson’ correlation coefficient, bias and limits of agreement.

**Results:** Pearson correlation coefficient, bias and limits of agreements between the CT non-aerated lung volumes and MRI abnormal lung volumes were 0.88, -16 ml, and (-108, 77), respectively. Pearson correlation coefficient between PaO_2_/FiO_2_ and abnormal lung volumes measured with MRI was -0.60.

**Conclusion:** In a preclinical swine model, quantitative measurements of pulmonary atelectasis by MRI-imaging are well correlated with the gold standard, i.e., densitometric scan CT measurements.

## Key Points

1. Question: Does MRI allow for reliable volumetric quantification of atelectasis?
2. Findings: Volume of atelectasis in an experimental perioperative lung aeration impairment model was well correlated between CT gold standard and MRI.
3. Meaning: MRI may be a radiation-free tool for quantification of perioperative atelectasis.

## Introduction

Pulmonary dysfunction is a common and potentially life-threatening complication after surgery (Nunn and Payne, 1962; Bendixen et al., 1963). Collapse of pulmonary alveoli, frequently referred to as atelectasis, has been reported to be one of the most frequent of these postoperative pulmonary complications (Fernandez-Bustamante et al., 2017). Suggested pathophysiological mechanisms for perioperative atelectasis include alveolar compression (Froese and Bryan, 1974; Hedenstierna et al., 1985; Fernandez-Bustamante et al., 2017), gas resorption in low ventilation/perfusion ratio lung zones (Duggan and Kavanagh, 2005), and surfactant impairment (Magnusson and Spahn, 2003). Atelectasis is associated with deleterious perioperative consequences, e.g., increased oxygenation requirement (Fernandez-Bustamante et al., 2017).

Computed-Tomography (CT) is an established and reproducible method of quantifying impairment of pulmonary aeration (Lundquist et al., 1995; Simon, 2000; Malbouisson et al., 2001). However, the significant radiation exposure associated with CT represents a limiting factor for its use in the perioperative setting. Indeed, ionizing radiation exposure during CT is up to 300 times the dose of a standard chest X-ray (Ludes et al., 2016). Magnetic resonance imaging (MRI), a radiation-free technique, is potential alternative to CT for atelectasis measurement (Ball et al., 2018; Kuethe et al., 2018). However, it has never been validated for the volumetric quantification of pulmonary aeration impairment (Duggan and Kavanagh, 2005).

Therefore, we conducted a preclinical study based on a perioperative atelectasis model to validate MRI based pulmonary aeration impairment quantification. We hypothesize that MRI based quantification of non-aerated lung volume is feasible and shows acceptable agreement with CT based measurements, the current gold standard.

## Materials and Methods

### Study Design

The study protocol was approved by our Institutional Review Board on December 2017 (#11615-201700212328528 v2) and care of the animals was provided according to the French law for experimental study and the ARRIVE guidelines (Kilkenny et al., 2010). The study took place in the Strasbourg Image-Guided Surgery Institute (Strasbourg University) from December 2017 to February 2018.

There was only one study group with each animal acting as its own control. All animals were treated according to the study design outlined in Supplementary Figure 1. Four time points were defined: prior to (T1) and after (T2) induction of general anesthesia and tracheal intubation, after 2 h of non-protective mechanical ventilation (T3), and after tracheal negative pressure application (T4).

### Animal Preparation

Before arriving in the experimental lab, *large white* pigs were sedated with subcutaneous tiletamine 100 mg – zolazepam 100 mg (Zoletil©, Virbac^TM^ laboratory) and azaperone 120 mg (Stresnil©, Elanco^TM^ laboratory), repeated once if required. After sedation was achieved, animals were weighed and then laid supine on the CT scan table (Somatom Definition©, Siemens Healthineers^TM^, Germany). A peripheral intravenous catheter was inserted in the ear and 0.9% saline was infused at 6 ml kg^-1^ h^-1^ during the procedure. An SpO_2_ monitoring probe was placed on the tail, and a rectal temperature probe was inserted in the rectum.

### Experimental Procedures

After induction of general anesthesia (intravenous propofol 2 mg kg^-1^ and rocuronium 1 mg kg^-1^) animals underwent orotracheal intubation (Intube©, Intersurgical^TM^). Non-protective mechanical ventilation was provided with an anesthesia station (Primus©, Dräger^TM^, Germany). Ventilation setting included volume mode with tidal volume set at 10 ml kg^-1^, zero end expiratory positive pressure, respiratory rate adjusted to aim at an end-tidal carbon dioxide expired pressure (PETCO_2_) between 35 and 45 mmHg. Fresh gas flow was set at 4 L min^-1^ with a 40% oxygen fraction (FiO_2_). Before the fourth time point (Supplementary Figure 1, T4), atelectasis was induced as previously reported (Strang et al., 2009; Schumann et al., 2011) by connecting the endotracheal tube to a Unimat 30©device (Karl Storz^TM^, Germany) and inducing a negative pressure of 20–80 kpa for 20 s). Animals were then ventilated as before for 40 s before the following time point.

Plateau pressure (Pplat) and calculated static lung compliance (Cpat) were recorded from the anesthesia station. Anesthesia was maintained with an infusion of propofol at 15 mg kg^-1^ h^-1^ and rocuronium at 1 mg kg^-1^ h^-1^. Arterial blood was collected by a femoral artery puncture before initiation of mechanical ventilation and through a femoral arterial catheter placed after initiation of mechanical ventilation. All arterial samples were analyzed with an on-site blood gas analyzer (Combi line©, Eschweiler^TM^, Germany).

### Imaging

At each time point (Supplementary Figure 1) a CT scan of the thorax was performed followed immediately by a MRI of the same region.

The CT images were acquired using a 64-row helical scanner (SOMATOM Definition AS©, Siemens Healthineers^TM^, Germany), after expiratory clamping of the tracheal tube for all intubated animals. Acquisition parameters were a tension of 80 kV and an automated tube current modulation maxed at 250 mAs. Images were reconstructed using a mediastinal (B31f) and a lung parenchyma (B70f) kernel at a slice thickness of 1.0 mm. The animals were then transported supine between the CT and MRI suites. Intubated animals were disconnected from the ventilator and no ventilation was performed during the 20 s transport.

The MRI images were acquired using a 1.5T MRI scanner (MAGNETOM Aera, Siemens Healthineers, Germany) with an 18-channel flex coil on the thorax and the integrated spine coil. A StarVIBE sequence was performed for T1 contrast. This sequence is a free-breathing radial 3D gradient-echo sequence. The acquisition variables were set as follows: echo time 1.88 ms, repetition time 4.08 ms, field of view 300 × 300 mm^2^, 1.2 × 1.2 mm^2^ in-plane resolution, flip angle 4°, 580 radial views and 176–224 1.3 mm thick slices depending on the size of the lungs. Acquisition times ranged from 225 to 287 s. The choice of a StarVIBE sequence over the more usual VIBE one (Spieth et al., 2018) was motivated by its isotropic voxel resolution allowing accurate 3D segmentation and its relative robustness to motion artifacts. A T2-weighted breath-hold TrueFISP sequence was also acquired with the following parameters: TE/TR = 1.43 ms/391 ms, in-plane resolution 0.8 × 0.8 mm^2^, 70 4-mm thick slices, acquisition time 27 s. This sequence was chosen for its short acquisition time. During the MRI, the animals were ventilated with an MRI safe ventilator (Fabius©, Dräger^TM^) using the same settings as during CT imaging.

**FIGURE 1:**
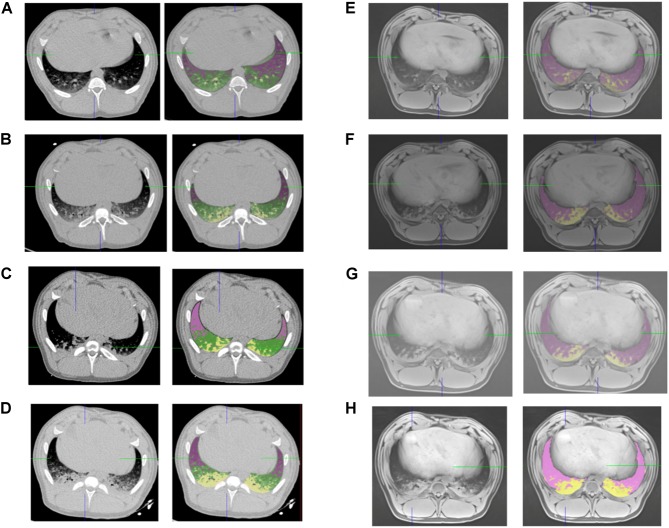
CT and MRI lung segmentations. Representative screenshot of the same subject lung segmentations based on CT (left panels) and MRI (right panels) before intubation **(A,E)**, after intubation **(B,F)**, after 2 h of mechanical ventilation **(C,G)**, and after induction of atelectasis with negative pressure **(D,H)**. Pink voxels: normal lung, yellow voxels non-aerated lung for CT and MRI, green voxels: poorly aerated lung for CT.

Measurements of lung volumes on CT and MRI images were performed by trained medical imaging technologists, using VP-Lab©software (Visible Patient©, Strasbourg, France). For CT scans, lung segmentation was performed using a color-encoding system based on CT radiodensity of voxels (Malbouisson et al., 2001): as represented in Figure 1, after exclusion of the mediastinum, voxels were defined as non-aerated lung (corresponding to atelectasis), poorly aerated lung, normally aerated lung and overinflated lung, according to their radiodensity, with respect of the following X-ray attenuation ranges: -100 to +100 HU, -500 to -100 HU, -500 to -900 HU and -1000 to -900 HU segments, respectively (Malbouisson et al., 2001). The total volume of each of the four lung compartments (non-aerated, poorly aerated, normally aerated and overinflated), classified as above and the total lung volume were then calculated by the software.

For MRI based lung aeration compartments measurements, was performed by color encoding based on voxel intensity. After exclusion of the mediastinum, voxels corresponding to non-aerated (i.e., atelectasis) or aerated lung were defined by a visual Voxel intensity value. The threshold intensity value corresponding to atelectasis was determined for each acquisition by visual confirmation of the superposition between color coding and T1-weighted hyperintense area (Figure 1). Color encoding and the non-aerated lung area was confirmed on T2 weighted images. The total volume of each of the two lung compartments (non-aerated and normally aerated) classified as above and the total lung volume were then calculated.

MRI-based lung segmentation cannot be based on signal intensity thresholds, since the reproducibility of signal intensity in MRI depends of the MRI scanner, the sequence used and the patient, and cannot be calibrated to a constant reference. Consequently, only a visual segmentation can be used and allows for two types of category (aerated and non-aerated lung parenchyma). On the contrary, CT voxel density numbers are highly reproducible between scanners and protocols (due to a calibration, where the air density is -1000 HU and pure water is 0 HU), and can be used to determine thresholds such as normally aerated, poorly aerated, non-aerated and overinflated.

The main objective of our study was to compare the non-aerated lung category (i.e., atelectasis) measured on both CT and MR images. The secondary endpoint was to compare the quantification of aerated lung as assessed by MRI with normally and poorly aerated lung assessed by CT, by hypothesizing that MR normally aerated lung would be correlated to CT normally + poorly aerated lung categories.

In each animal, CT-based and MRI-based lung compartment measurements were performed by the same medical imaging technologists. The accuracy and relevance of the color coding segmentation on CT and MRI images was confirmed by a board-certified radiologist. The radiologist also analyzed the CT scans to rule out pleural effusion.

## Sample Size

This study was a pilot validation study and no preliminary data were available. Therefore, we based our sample size on a similar studies design (Acosta et al., 2014; Ball et al., 2017a). We aimed at measuring at least 30 pairs of CT/MR volume measurements and planned to increase the total number of measurements to 40 to replace technical failures.

## Analysis Plan

The main objective of the study was to assess the experimental validation of MRI to measure perioperative pulmonary aeration impairment such as non-aerated lung volumes (atelectasis) (Lundquist et al., 1995). To validate MRI-based pulmonary aeration impairment we aimed at confirming two hypotheses: (1) MRI non-aerated lung quantification would correlate with the gold standard CT non-aerated lung measurement and (2) MRI non-aerated lung measurement would negatively correlate with arterial oxygenation.

## Statistical Analysis

Continuous variables are presented as mean ± SD. Repeated data were analyzed using mixed models with a fixed time effect and a random subject effect (package hglm2 in R software). Bland and Altman (1999) plots were used to estimate the between methods agreement. Correlations between continuous variable were computed using Pearson correlation coefficient. A *p*-value less than 0.05 was considered as statistically significant. No correction for multiple comparisons was applied. All computation were done with R 3.3.1 (R Core Team, 2016).

## Results

Ten animals were studied. The mean body weight was 37.8 ± 5.6 kg. Overall, 40 CT scans and 34 MRI scans were included in the analysis. Six MRI scans at the first time point, “before intubation,” were not analyzed because of motion artifact induced by animal movement. Plateau pressure and compliance values were not recorded in the first four animals for technical reasons. Physiological and blood gas measurements are represented in Table 1.

**Table 1:** Physiological and blood gas measurements.

	Before intubation	After intubation	2 h mechanical ventilation	Tracheal negative pressure
SpO_2_ (%)	93 (6)	97 (4)^*^	99 (2)^*^	92 (8)
PETCO_2_	NA	41 (6)	37 (6)^#^	39 (5)
Heart rate (Bpm)	86 (22)	107 (37)^*^	87 (23)	90 (24)
Mean arterial pressure (mmHg)	NA	54 (13)	62 (7)	61 (13)
T° (°C)	36.6 (0.8)	36.5 (0.8)	36.3 (1.4)	35.7 (1.7)
**Arterial blood gas**				
pH	7.48 (0.05)	7.41 (0.09)^*^	7.46 (0.07)	7.41 (0.08)^*^
PaO_2_ (mmHg)	74 (18)	161 (44)^*^	169 (39)^*^	111 (48)^*^
PaCO_2_ (mmHg)	42 (7)	52 (14)^*^	46 (9)	55 (12)^*^
HCO_3_ (mmol l^-1^)	30 (3)	31 (5)	32 (2)	33 (2)^*^
PaO_2_/FiO_2_ (mmHg)	352 (85)	403 (110)	423 (99)^*^	278 (121)^*^

*^∗^p < 0.05 vs. before intubation, ^#^p < 0.05 vs. after intubation. Data are presented as mean and standard deviation (SD), NA = not applicable.*

**FIGURE 2:**
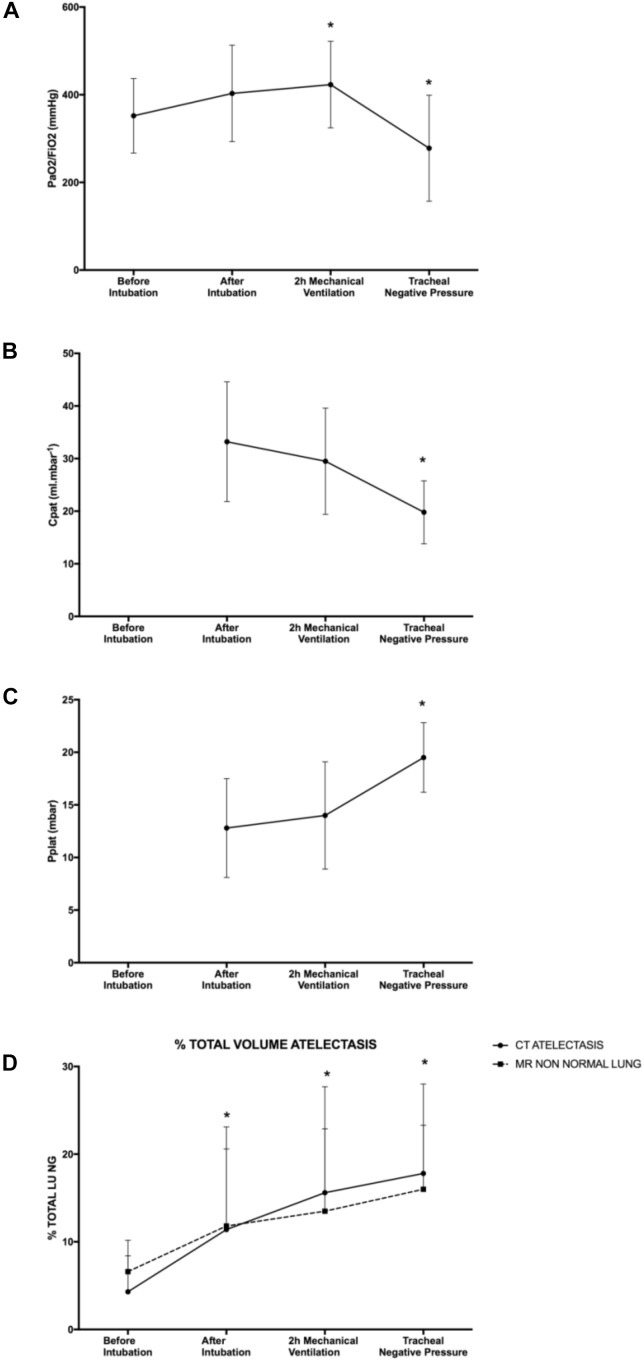
Respiratory function. **(A)** PaO_2_/FiO_2_ measurement, **(B)** patient airway compliance, **(C)** plateau pressure, and **(D)** percentage of total lung volume measured as non-aerated lung on CT scan and MRI. Plateau pressure and patient airway compliance values before intubation were not available. Mean with SD. ^∗^*p* < 0.05 compared to before intubation for **(A,D)** and compared to after intubation for **(B,C)**.

Oxygenation and ventilation characteristic are represented in Table 1 and Figure 2.

Baseline (T1) PaO_2_/FiO_2_ was 352 ± 85 mmHg, increased to 423 ± 99 mmHg (*p* = 0.020) after 2 h of mechanical ventilation and dropped to 278 ± 121 mmHg (*p* = 0.016) after application of intra-tracheal negative pressure. Compared with the time point “after intubation,” after tracheal negative pressure application, pulmonary compliance decreased (20 ± 6 vs. 33 ± 11 ml mbar^-1^, *p* = 0.00027) and plateau pressure increased (20 ± 3 vs. 13 ± 5 mbar, *p* = 00014) significantly.

By CT imaging (gold standard), non-aerated lung volumes significantly increased after tracheal intubation (11.4 ± 9.2% of total lung, *p* = 0.002), after 2 h of mechanical ventilation (15.6 ± 12.1% of total lung, *p* = 0.00001) and after application of intra-tracheal negative pressure (17.8 ± 10.2% of total lung, *p* = 0.0000006) compared to baseline (4.3 ± 5.9% of total lung, Table 2). Results were similar for MRI-based imaging: non-aerated lung volumes measurements increased significantly after tracheal intubation (11.8 ± 11.3% of total lung, *p* = 0.03), after 2 h of mechanical ventilation (13.5 ± 9.4% of total lung, *p* = 0.006) and after tracheal depression (16.0 ± 7.3% of total lung, *p* = 0.0005) compared to baseline measurement (6.6 ± 1.8% of total lung). None of the animals developed pleural effusion.

**Table 2:** Lung segmentation based on CT and MR measurement.

		Before intubation		After intubation		2 h mechanical ventilation		Tracheal depression	

		**ml**	**% of total volume**	**ml**	**% of total volume**	**ml**	**% of total volume**	**ml**	**% of total volume**
**CT lung segmentation**									
Non aerated		54 (71)	4.3 (5.9)	113 (87)^*^	11.4 (9.2)^*^	148 (101)^*^	15.6 (12.1)^*^	175 (85)^*^	17.8 (10.2)^*^
Poorly aerated		373 (75)	30.1 (9.7)	361 (86)	35.3 (11.0)^*^	316 (62)^*^	31.7 (7.9)	298 (89)^*^	29.5 (9.8)
Normally aerated		880 (347)	65.2 (13.4)	578 (274)^*^	53.0 (19.4)^*^	553 (250)^*^	52.5 (18.4)^*^	567 (287)^*^	52.4 (18.7)^*^
Overinflated		5 (4)	0.35 (0.25)	4 (3)	0.31 (0.18)	3 (2)^*^	0.29 (0.13)	4 (4)	0.36 (0.26)
Total volume		1315 (278)	–	1055 (184)^*^	–	1020 (161)^*^	–	1045 (199)^*^	–
**MR lung segmentation**									
Non-aerated		69 (24)	6.6 (1.8)	134 (112)^*^	11.8 (11.3)^*^	158 (110)^*^	13.5 (9.4)^*^	185 (74)^*^	16.0 (7.3)^*^
Aerated		965 (158)	93.4 (1.8)	1084 (262)	88.2 (11.3)^*^	1031 (191)	86.5 (9.4)^*^	1009 (199)	84.1 (7.3)^*^
Total volume		1034 (174)	–	1218 (196)	–	1189 (148)	–	1193 (160)	–

*^∗^p < 0.05 compared to before intubation measurement, ml = milliliter. Data are presented as mean and standard deviation (SD).*

**FIGURE 3:**
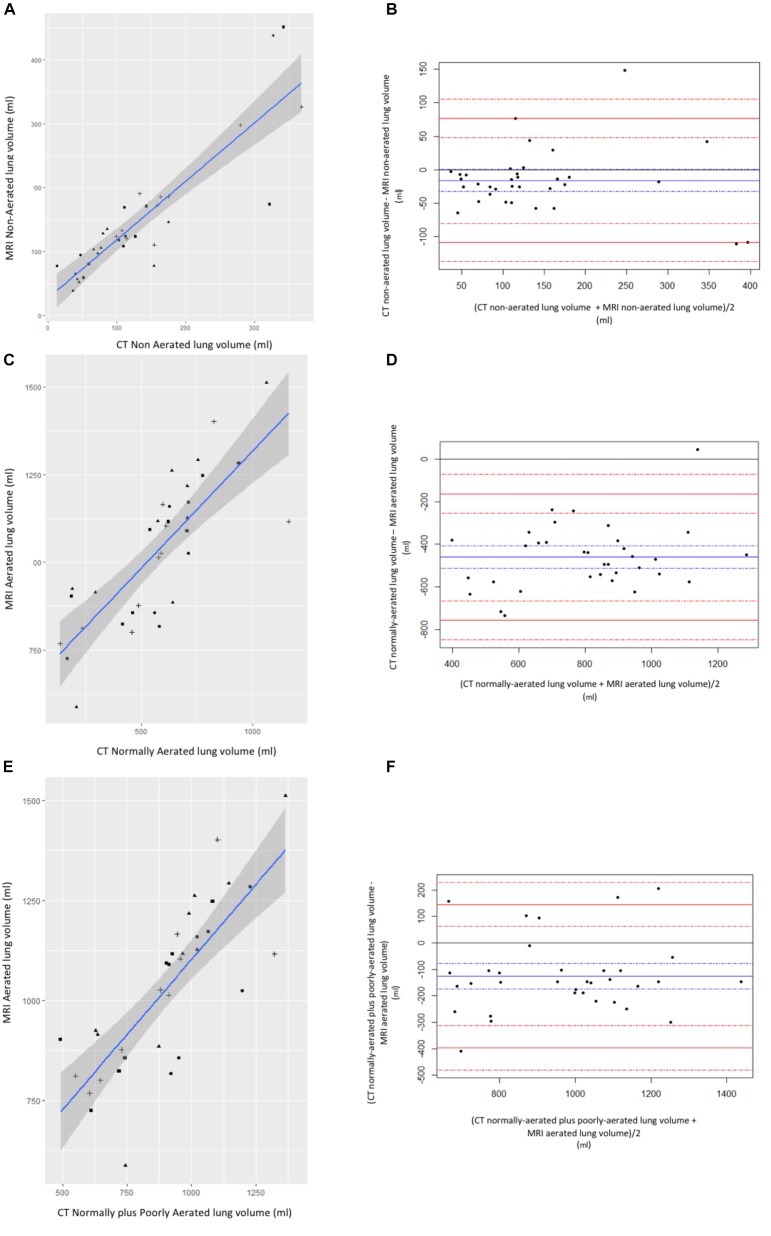
Correlation between CT and MR lung volumes. **(A)** Correlation and **(B)** Bland–Altman plotting between non-aerated lung volume measured on CT and non-aerated lung volume measured on MRI. **(C)** Correlation and **(D)** Bland–Altman plotting between normally aerated lung volume measured on CT and normally aerated lung volume measured on MR. **(E)** Correlation and **(F)** Bland–Altman plotting between normally plus poorly aerated lung volume measured on CT and normally aerated lung volume measured on MRI. Circle: before intubation timepoints; triangle: after intubation time points; square: 2 h ventilation time points; cross: tracheal depression time points. Left panels: blue line is for linear regression slope and gray zone for 95% confidence interval. Right panels: blue line is for bias and dashed blue lines for 95% confidence interval margins; red lines are for limits of agreements and dashed red lines for their respective 95% confidence interval margins.

Correlation and Bland Altman plotting between CT and MRI-based lung segmentation volumes are represented in Figure 3. Pearson correlation coefficient, bias and limits of agreement were 0.88, -16 ml, and (-108, 77), respectively, between CT-based vs. MRI-based non-aerated lung volumes. Pearson correlation coefficient, bias and limits of agreement were 0.79, -460, and (-757, -163), respectively, between CT-based vs. MRI-based normally aerated lung volumes. Last, Pearson correlation coefficient, bias and limits of agreement between CT-based normally aerated lung volumes plus poorly aerated lung volumes and MRI-based aerated lung volume, were 0.79, -126 ml, and (-397, 145), respectively.

**FIGURE 4:**
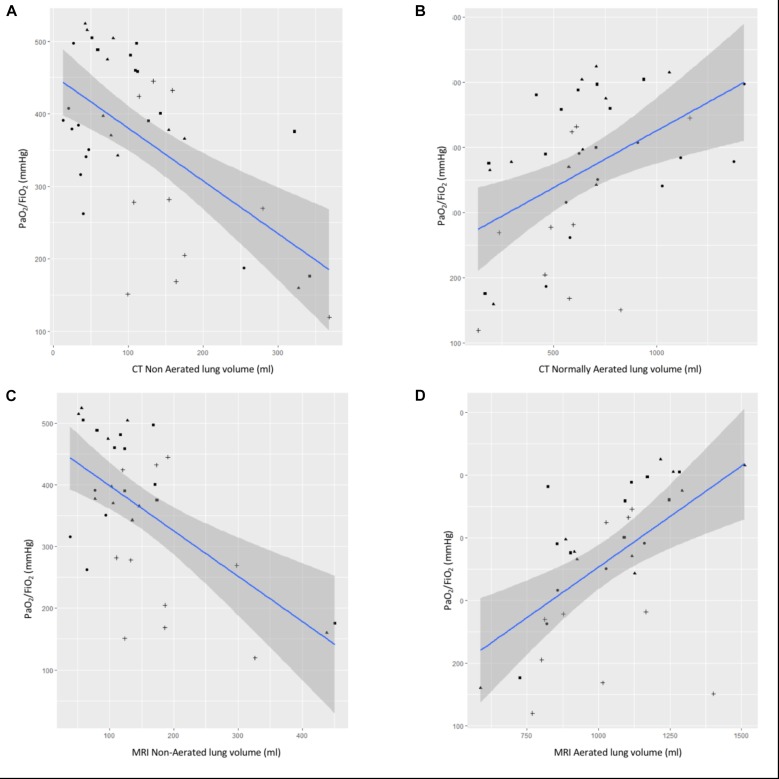
Correlation between CT or MR lung volumes and PaO_2_/FiO_2_. Correlation between PaO_2_/FiO_2_ and **(A)** CT non aerated lung volume, **(B)** CT normally aerated lung volume, **(C)** MRI non-aerated lung volume, and **(D)** MRI normally aerated lung volume. Blue line is for linear regression slope and gray zone for 95% confidence interval.

Correlations between aerated, non-aerated CT-based lung volumes or aerated, non-aerated MRI-based lung volumes and PaO_2_/FiO_2_ ratio are represented in Figure 4. Pearson correlation coefficient between PaO_2_/FiO_2_ ratio and CT-based non-aerated lung volumes or MRI-based non-aerated lung volumes were -0.60 or -0.60, respectively. Pearson correlation coefficients comparing PaO_2_/FiO_2_ and normally aerated lung volumes measured with either CT or MRI were 0.47 and 0.56, respectively.

## Discussion

The main result of our study is the validation of MRI to quantify non-aerated lung volumes, i.e., atelectasis, compared with the gold standard of CT. This validation is supported by: (1) very good correlation between MRI-based and CT-based non-aerated lung volume measurements (Spearman coefficient: 0.88; bias: -16 ml; and limits of agreement: -108–77 ml), and (2) the negative correlation between MRI-based non-aerated lung volumes and PaO/FiO_2_ (Spearman coefficient: -0.6). Our results suggest that the volume of non-aerated lung identified on MRI images corresponds to the volume of non-aerated lung according by CT scan (gold-standard) which was our primary endpoint. We focused on this category because it may be clinically relevant for patients in the perioperative setting (Duggan and Kavanagh, 2005).

Our results also suggest that the volumes of lung identified as aerated on the MR images tends to correspond to the volumes of normally and poorly aerated lung according to the CT scan gold standard segmentation. This summation of 2 CT lung aeration threshold (normally and poorly aerated) corresponding to one MRI category (aerated lung) is explained by the fact that the MR image signal scale only allows for visual identification of two different types of lung signal. The CT references method is described with four different types of lung attenuation thresholds (non-aerated, poorly aerated, normally aerated, and overinflated). That is the reason why we aimed, as a secondary endpoint, at describing correspondence of MR aerated lung to CT categories (normally plus poorly aerated lung volumes).

Quantitative assessment of lung aeration based on MRI has been very limited, mainly because of technical challenges of MR imaging (Ball et al., 2017b), for example, the prolonged time required for image acquisition, and the low proton density within the lung parenchyma. However, recent experimental data, *ex vivo* (Ball et al., 2018) and *in-vivo* (Kuethe et al., 2018) have suggested that lung MRI may be feasible and produce clinically valuable information (Ozcan et al., 2017), including functional imaging (Mendes Pereira et al., 2018). Ball et al. observed in *ex-vivo* pig lungs that lung density was a linear function of MRI T2 signal intensity normalized to muscle intensity. This work proposes an interesting way to possibly overcome the issues associated non-calibrated MRI signal intensity by normalizing to adjacent tissue like muscle (Ball et al., 2018). We believe that our results are complemental to this study. In the previous study from Dr Ball, the main objective was to assess the ability of MRI to detect differences in lung aeration in an ex-vivo porcine model. The authors also reported the feasibility of MRI based atelectasis measurement in a clinical setting. Our study offers complementary results. First, our imaging measurements were performed *in vivo*. *In vivo* lung assessment is important because of the potential artifacts induced by the surrounding tissue environment of the lungs. Second, our study included CT-scan based lung aeration measurements, considered the clinical gold-standard.

The MRI sequence we used for our study was relatively short (4–5 min) and used a motion-robust 3D sampling scheme allowing for free-breathing acquisition. Tusman et al. (Tusman et al., 2003; Acosta et al., 2014) reported a semi-quantitative method of measuring lung atelectasis with MR in anesthetized children. Briefly, the authors studied two axial MRI slices that were divided into six equal sectors radiating at identical angles from the center of the thorax image. Each of the twelve sectors was then analyzed for the absence or presence of atelectasis. This interesting method, however, has several limitations: (1) it is not completely quantitative, (2) it does not assess the whole lung, and (3) it has not been validated compared to the gold standard of CT scan.

Other radiation free methods have potential for the diagnosis of perioperative atelectasis. First, lung ultra-sound may be a useful, bedside, lung aeration assessment tool. Acosta (Acosta et al., 2014) reported interesting results in a pediatric population where lung ultrasound was used to diagnosis of atelectasis. However, this method (Acosta et al., 2014) is largely semi-quantitative, as it does not allow for atelectasis volume measurement and examination of the entire lung is often not feasible due to the absence of ultrasound propagation across lung air. Second, electrical impedance tomography (EIT) has been reported to assess changes in lung aeration during acute respiratory distress syndrome treatment (Mauri et al., 2016). However, EIT technology only explores one slice of an individual thorax, with a low spatial resolution. These three radiation-free technologies (i.e., MRI, ultrasounds, and EIT) might complement one another for assessment of lung aeration in the perioperative setting: ultrasound is a useful bedside diagnostic tool, EIT offers continuous monitoring of ventilation distribution, and MRI allows for complete lung exploration and quantification of atelectasis. Moreover, images captured by MRI can be stored and compared to subsequent evaluations.

Strategies to reduce the dose of radiation have been proposed to overcome the quantitative assessment of lung aeration using conventional whole lung CT scan. For example, Chiumello et al. assessed quantitative aeration in ARDS patients with 110, 60, and 30 mAs acquired CT scans. Correlation of measured atelectasis was excellent (*r*^2^ at 0.99 for both 110-60 and 110-30 mAs groups) with limited bias (-0.1 and -0.2 for the 110-60 and 110-30 mAs groups). In a translational study, Ball et al. (2017a) compared, quantitative assessments of lung aeration by standard spiraled whole lung CT or ultra-low dose sequential slice CT acquisition in swine and humans. The author reported good agreement between standard and ultra-low dose techniques. All biases were lower than 2% for segmented aeration compartments and 10 ml for total volume. However, the median effective dose of an ultralow dose CT assessment was still at 0.09 mSv which is 5% of the annual dose limit recommended by the International Commission on Radiation Protection (2007). Additionally these studies were based on ARDS-associated lung aeration impairment and these result may not be generalizable to the perioperative setting. Availability of a validated, radiation free quantitative measurement of lung atelectasis may allow for easier diagnostic and treatment monitoring of pulmonary dysfunction in certain patients. The need for more research on MRI-based lung aeration assessment has been emphasized by some experts in the field (Duggan and Kavanagh, 2005; Ball et al., 2017b).

Our study has several limitations. First, it is a preclinical study. Therefore, these results will need to be translated and validated into a clinical setting. It is reassuring that swine models of acute lung injury and acute respiratory distress syndrome have been used extensively and may be more relevant to clinical practice than other preclinical models, e.g., rodent (Ballard-Croft et al., 2012). Second, animals were moved from the CT scan table to the MRI table between each acquisition. This movement may have affected the degree of lung aeration measured by CT vs. MRI To minimize this potential impact, we transferred the animals supine, under expiratory apnea to avoid any recruitment induced with manual bag ventilation, and in a very fast process (<30 s). Third, most of the MRI acquisitions were done under general anesthesia and mechanical ventilation, where the breathing is regular and smooth. it is possible that image quality in a spontaneously breathing patients will be worse due to irregular breathing patterns. Fourth, our model did not produce significant overinflated lung volume, therefore we could not assess the use of MRI for this lung pathology. Our model included negative tracheal pressure application as already reported in another preclinical perioperative atelectasis model (Strang et al., 2009). This model may not reflect all etiologies of perioperative atelectasis. The disconnection of the animal from the respiratory circuits for the transfer between MRI and CT scan suites may also have modified the amount of atelectasis and induced systematic errors. In addition, transporting mechanically ventilated patients to the MRI suites is much more complicated as compared to CT-scan. However, obtaining a MRI is less complicated for non-intubated patients. MRI scans may also be increasingly available in the operating room as image-guided surgery expands. The feasibility of perioperative MRI-based measurement of atelectasis has already been used by Spieth et al. (2018) in a clinical trial. Perioperative respiratory medicine could benefit from an increased availability of MRI.

## Conclusion

In a preclinical swine model, quantitative measurements of pulmonary atelectasis by MRI-imaging are well correlated with the gold standard, i.e., densitometric scan CT measurements. Prospective studies in humans are needed to address whether MRI, a radiation-free tool, can be used to help diagnose and manage patients with atelectasis in the perioperative period.

## Ethics Statement

The study protocol was approved by our Institutional Review Board on December 2017 (#11615-201700212328528 v2) and care of the animals was provided according to the French law for experimental study and the ARRIVE guidelines. The study took place in the Strasbourg Image-Guided Surgery Institute (Strasbourg University) from December 2017 to February 2018.

## Author Contributions

EN designed and conducted the study, analyzed the results, and wrote the manuscript. MO designed the study, analyzed the results, and reviewed the manuscript. MH conducted the study and reviewed the manuscript. EB-G analyzed the results and reviewed the manuscript. MD designed the study and reviewed the manuscript. CG designed and conducted the study, and reviewed the manuscript. JP reviewed the manuscript. NM analyzed the statistical data and reviewed the manuscript. PD designed the study and reviewed the manuscript.

## Conflict of Interest Statement

The authors declare that the research was conducted in the absence of any commercial or financial relationships that could be construed as a potential conflict of interest.
