# Supplementary material for: Validation of MRI for Volumetric Quantification of Atelectasis in the Perioperative Period: An Experimental Study in Swine
Source: Front Physiol. 2019 Jun 4;10:695. doi: 10.3389/fphys.2019.00695 (PMC6558191; doi:10.3389/fphys.2019.00695)
Supplement: Supplementary file 1 [file Image_1.pdf]

Supplemental Figure S1: Experimental Measures Time Schedule

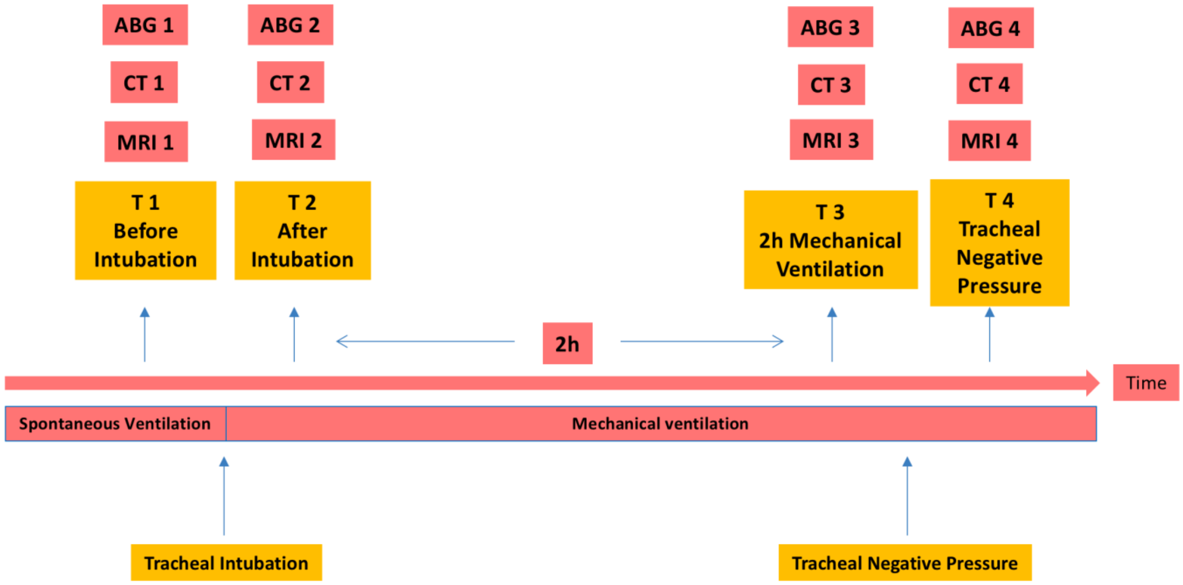

ABG: Arterial Blood Gas, CT: Computed Tomography, MRI: Magnetic Resonance Imaging.
